# Forty-Year Long-Term Outcome After Endoscopic and Open Surgery for Esthesioneuroblastoma in Consideration of Prognostic Factors

**DOI:** 10.3390/cancers17030343

**Published:** 2025-01-21

**Authors:** Michael Koch, Matthias Balk, Sven Schlaffer, Abbas Agaimy, Heinrich Iro, Sarina K. Mueller

**Affiliations:** 1 Department of Otorhinolaryngology and Head and Neck Surgery, University of Erlangen–Nuremberg, 91054 Erlangen, Germany; matthias.balk@uk-erlangen.de (M.B.); heinrich.iro@uk-erlangen.de (H.I.); sarina.mueller@uk-erlangen.de (S.K.M.); 2 Department of Neurosurgery, University of Erlangen–Nuremberg, 91054 Erlangen, Germany; sven.schlaffer@uk-erlangen.de; 3 Institute of Pathology, University of Erlangen–Nuremberg, 91054 Erlangen, Germany; abbas.agaimy@uk-erlangen.de

**Keywords:** craniofacial, endoscopic, esthesioneuroblastoma, transcranial, bicoronar, transfacial, surgery

## Abstract

**Background:** The objective of this study was to compare results after endoscopic (ES) and open surgery (OpS) for esthesioneuroblastoma over a 40-year period. **Methods:** In a retrospective study, patients who had undergone ES and OpS for esthesioneuroblastoma with curative intent were included. The following outcome parameters were compared after ES and OpS: epidemiologic, clinical (including known tumor classifications), histopathologic, therapeutic (resection state, adjuvant therapy), and development of recurrences. Crude survival and Kaplan–Meier 10 y, 20 y, and 35 y actuarial survival were calculated. **Results:** Between 1981 and 2021, 15 patients were operated with ES and 28 with OpS. Advanced-stage tumors were significantly more often treated using OpS. For all other parameters, there were no other significant differences between ES and OpS. After ES, the 35 y OS, DSS, and DFS were 48.1%, 100%, and 55.9%, respectively. After OpS, they were 40.5%, 77.5%, and 35.3%. **Conclusions:** ES is an effective approach in esthesioneuroblastoma, even in the long-term course over decades, if the appropriate indication compared to OpS is respected.

## Simple Summary

Therapy in esthesioneuroblastoma (ENB) involves complete surgical resection with or without adjuvant therapy. Complete resection can be achieved by various surgical methods including various open approaches (OpS) and endoscopic-controlled surgery (ES). During the last two decades minimal invasive endoscopic-controlled surgery was introduced and established. OpS and ES were compared in various reports and ES proved to be of equal value at least for smaller tumors. However, data comparing long-term results after OpS and ES are missing. In this manuscript, we compared results and survival after OpS and ES over four decades. We found in our report that ES, when the limitations of this approach are respected, can be performed like OpS with an acceptable outcome also in the long-term course. Our publication confirms that successful management of ENB is possible by less invasive surgery.

## 1. Introduction

Treatment for esthesioneuroblastoma (ENB) consists of complete surgical resection, as well as adjuvant therapy depending on risk factors and tumor stage [1,2,3].

Craniofacial resection (CFR) was long regarded as the gold standard in the treatment of ENB [4,5,6,7,8,9,10,11,12,13,14,15]. Endoscopy-assisted CFR (CFR + E-ass) was later introduced [11,15,16,17]. Results after transcranial/bicoronar resection with or without endoscopic assistance (BCR/TCR ± E-ass) [5,18,19,20] and after transfacial resection (TFR) [4] or endoscopy-assisted TFR (TFR + E-ass) [5,13,14,21] were also published. A shift toward purely endoscopic surgery (ES) took place in the early twenty-first century [22,23], and numerous publications followed [24]. The results after ES were compared with open surgery (OpS) in some reports, to assess the value of ES [15,25,26,27,28,29].

As stage is considered a significant prognostic factor [30,31], various classifications have been described, such as those published by Kadish [32], Morita [33], Dulguerov and Calcaterra [4], Koka [34], Biller [35], and Resto [36]. The Hyams grade has been recognized as an important prognostic factor [30,31,37]. The Ki-67 labeling index, discussed in only a few reports, is considered another potential prognostic factor [38].

The aim of the present study was to compare the results and outcomes after ES or OpS with curative intent in our department, taking into account various prognostic factors over a long-term course of 40 years.

## 2. Materials and Methods

This retrospective study was carried out at the Department of ENT, Head and Neck Surgery of Friedrich–Alexander University of Erlangen–Nuremberg. The study was conducted in full accordance with ethical principles, including the World Medical Association Declaration of Helsinki (version 2002). Approval for the study was obtained from the local institutional review board of the FAU Erlangen–Nuremberg (no. 20-292-Br).

The hospital database and clinical reports were searched for patients who had undergone surgical treatment for ENB with curative intent in the department between 1981 and 2021. All of the patients received regular follow-up. If the patients were also treated during the further course of the disease in another institution, reports were requested and data were evaluated. If presentation for follow-up was not desired or possible in the further follow-up, reports from the patient’s treating physician/hospital were requested and evaluated, and the patient was contacted by phone.

The epidemiologic data assessed were age, age <50 vs. >50 years, and gender. Preoperative and retrospective staging of tumors was carried out in accordance with the classifications of Kadish [32], Morita [33], Dulguerov and Calcaterra [4], Koka [34], Biller [35], and Resto [36]. OpS was performed as a TFR (Weber–Ferguson ± modification following Janaczek) or BCR/TCR. ES was performed using 30–70° angled nasal endoscopes with navigation control. Additional parameters assessed were state of resection margins (negative vs. positive/close/unknown), postoperative complication rates, application of adjuvant/multimodal therapy (radiotherapy/chemoradiotherapy ± biologicals), radiotherapy dosage, Hyams grade (values, low/high-grade), Ki-67 labeling index (values <10% versus >10%), and recurrences (number/time after surgery/location).

The end points of the study were primary tumor progression, tumor recurrence, overall survival (OS), disease-specific survival (DSS), and disease-free survival (DFS). The patients’ survival status was calculated from the time of first presentation to last contact using the crude OS, DSS, and DFS and actuarial OS and DSS in accordance with the Kaplan–Meier method. DFS according to Kaplan–Meier was defined as time to first recurrence and/or tumor progression, which included also development of metastases or death of any cause after curative therapy.

### Statistical Analysis

SPSS Statistics for Windows, version 26 (IBM Corporation, Armonk, NY, USA), was used for all analyses. All data are given as mean ± standard error of the mean (SEM), median, and range. Bivariate correlations were calculated using the Pearson correlation coefficient. Differences between the groups for continuous variables were calculated using the Mann–Whitney U-test. Differences and associations between the groups for categorical variables were calculated using the Pearson chi-squared test with an exact 2-sided significance. Survival analysis was performed using the Kaplan–Meier method. Differences between groups were calculated using the log-rank test. The significance level was set at *p* ≤ 0.05.

## 3. Results

### 3.1. Preoperative Clinical Data and Tumor Stage

Forty-three patients with a mean age of 52 years were included. Of them, 67.4% were >50 years of age, and 51.2% were male. No differences were noted between the ES and OpS groups. Advanced tumor stages at first presentation were Kadish C/Morita C–D in 44.2%, Dulguerov and Calcaterra T3–4 in 41.9%, Koka T3–4 in 51.2%, and Biller/Resto ≥ T2 in 55.8%. Advanced tumor stages were significantly more often noted in patients treated with OpS (*p* = 0.004; *p* = 0.009; *p* = 0.01; *p* = 0.0001). No primary lymph node or distant metastases were present at first diagnosis (Table 1 and Table 2).

### 3.2. Surgical Treatment, Postoperative Complications, and Adjuvant Therapy

ES was performed in 34.9% of the patients, and NMs were achieved in 86.7%. Combination therapy was performed in 53.3% and multimodal therapy in 6.7%. Surgical complications were noted in 13.3% (2/15: one cerebrospinal fluid fistula (CSF), one CSF + hematoma with temporary signs of hemiplegia; Table 2b).

OpS was performed in 65.11% of the patients. Negative margins (NMs) were achieved in 64.29%, in 33.3% (1/3) after TFR, and in 68.0% (17/25) after TCR. Postoperative complications were observed in 32.1% (9/28: one intracranial hematoma + epilepsy; one epilepsy; one diabetes insipidus; two patients with disturbed wound healing and consecutive bone defect in the skull base; three patients with CSF leaks; one patient with loss of vision after an orbital hematoma). Combined treatment was performed in 84% (21/25) and multimodal therapy in 4% (1/25). No significant differences between the ES and OpS patients were observed in relation to these parameters, including the radiotherapy (RT) dose (Table 2b).

### 3.3. Histopathological Data

Hyams grading was assessed in 40 cases, 93% of all cases (40/43): grade I, n = 8 (20.0% of assessed cases), grade II, n = 15 (37.5% of assessed cases), grade III, n = 16 (40.0% of assessed cases), and grade IV, n = 1 (2.5% of assessed cases). Among the available ES patients, 35.7% had high-grade tumors, in comparison with 46.2% of the available OpS patients. The Ki-67index was assessed in 36 cases, 83.72% of all patients (36/43; mean 13.24 ± 1.72). Twenty-one patients (58.3% of assessed patients) had a value of >10%. Among the ES patients assessed, 46.2% had a Ki-67 index ≥10%, in comparison with 65.2% of the OpS patients assessed. No significant differences were calculated when the values themselves were compared. The same was also the case if tumors with a Ki-67 of <10% versus >10% (*p* = 0.31) and tumors with Hyams grades I–II versus III–IV (*p* = 0.74) in the ES and OpS patients were compared (Table 2b).

### 3.4. Postoperative Clinical Course, Survival, and Outcome Analysis

Primary tumor progression was observed in one patient after OpS. This patient had extensive disease (Kadish/Morita C, Dulguerov and Calcaterra T4, Koka T4, Resto T3), Hyams grade III, and a Ki-67 value of 26.5%. The patient died of the disease after 4 months.

Recurrence rates and metastases were noted in 37.2% (16/43) of all patients (five local, six regional, three distant, one local + regional, one regional + distant).

After ES, recurrences were observed in 26.7% (4/15: one locoregional, two regional, and one regional + distant recurrence) and after OpS in 42.9% (12/28: five local, four regional, three distant; *p* = 0.34). The first recurrence appeared after a mean of 86 months after ES and 65 months after OpS (*p* = 0.45). The total numbers of recurrences after ES were n = 1 in three patients (20%) and n = 3 in one patient (6.7%), and after OpS n = 1 in five patients (17.9%), n = 2 in three patients (10.7%), and n = 3 in one patient (3.6%). No significant differences between ES and OpS were calculated with regard to recurrence rates (*p* = 0.34), time to first recurrence (*p* = 0.86), or number of recurrences/patient (*p* = 0.72). The time to first recurrence was not significantly associated with OS, DSS, or DFS for all cases (*p* = 0.25; *p* = 0.28; *p* = 0.79) and also for the ES group (*p* = 1.0 each) and the OpS group (*p* = 0.47; *p* = 0.47; *p* = 0.59). Whenever possible, one or several salvage therapies with curative intent were performed in 81.25% of all patients with recurrences (13/16). A more detailed analysis of the salvage therapy, however, is beyond the scope of this manuscript.

Altogether, tumors with unfavorable criteria or course (Ki-67 index > 10%, Hyams grades III–IV, extensive tumor, tumor progression, recurrence) were present in 41.86% of all cases, in 33.33% of ES cases, and in 46.42% of OpS cases. No significant differences were noted between the ES and OpS groups (*p* = 0.52; Table 2b).

The follow-up period for patients with ES was 139 months (range 9–494 months) and for OpS patients 174 months (range 4–440 months), with no significant differences between the two groups (*p* = 0.646).

Analysis of the crude survival for ES and OpS provided crude survival rates for OS (73.3% vs. 50%, *p* = 0.199), DSS (100% vs. 78.6%, *p* = 0.076), and DFS (66.7% vs. 53.7%, *p* = 0.523). All of these rates were slightly higher after ES, but with no significant differences in comparison with OpS. Nevertheless, a trend toward better survival after ES for DSS and DFS was recognizable (Table 3).

**Figure 1:**
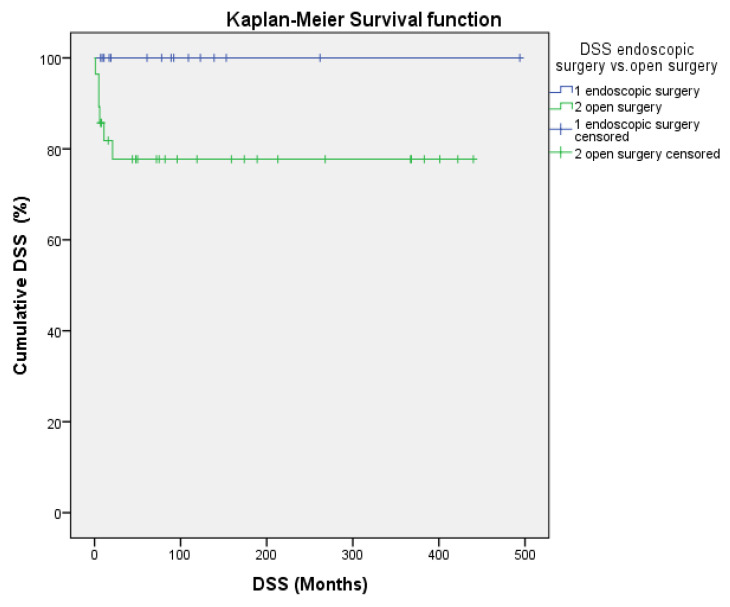
10-, 20-, and 35-year DSS according to Kaplan–Meier after ES (1, blue color) and OpS (2, green color) of ENB (*p* = 0.071).

**Figure 2:**
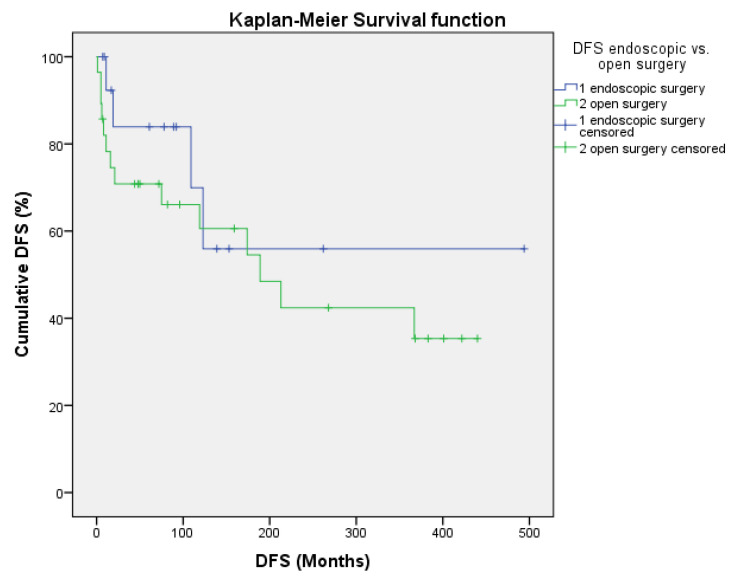
10-, 20-, and 35-year DFS according to Kaplan–Meier after ES (1, blue color) and OpS (2, green color) of ENB (*p* = 0.402).

Kaplan–Meier actuarial survival was calculated for 10, 20, and 35 years (Table 3). The 35 y OS, DSS, and DFS rates for all patients were 43.3%, 84.9%, and 43.3% and for the ES group, they were 48.1%, 100%, and 55.9%, respectively. The mean 35 y OS period was 311 months. For the OpS group, they were 40.5%, 77.5%, and 35.3%. The mean 35 y OS period was 248.4 months, with no significant differences from the ES group (*p* = 0.40). The 10 y, 20 y, and 35 y DSS rates were all 100% after ES and all 77.5% after OpS, with a trend toward a better DSS for the ES group (*p* = 0.071; Figure 1). Altogether, no significant differences between ES and OpS groups were noted when any 10 y, 20 y, or 35 y actuarial survival data or mean survival periods were compared (Figure 1 and Figure 2, Table 3).

## 4. Discussion

Since the end of the 1990s, it has been shown that piecemeal resection is not a disadvantage in comparison with en bloc resection in malign sinonasal tumors, including ENB, and that ES can be performed, taking its limitations into account, with acceptable results comparable to those with OpS in terms of complication rates, survival rates, and recurrence rates [11,12,15,22,23,39,40,41,42,43,44,45,46]. Successful ES has also been reported in patients with advanced tumor stages (Kadish C/D and/or T3–4) in up to 50–70% of cases in some reports [15,23,40,41,42,43,44,45,46,47].

Results after ES in patients with ENB were summarized in a recent systematic review that included 44 studies with 399 patients. On average, 48.3% of the patients had advanced stages (mod. Kadish C/D), while 34% had Hyams grades III–IV. NMs were achieved in 86.9%. Adjuvant RT was administered in 83%. The mean recurrence rate was 10.3%, with a mean time to recurrence of 56.6 months (range 7–192 months). The mean reported follow-up period was 53.5 months, with a range of 3–242 months. In patients with a 5 y follow-up, the mean 5 y OS was 91.1% [24].

OpS was investigated in one earlier meta-analysis that included 26 studies, comprising 390 patients, published between 1990 and 2000 [6]. A mean of 61% of the tumors were Kadish C stage, and 50% were staged T3–4. Hyams grades III–IV were present in 38%. Surgery and RT (44%; RT dose 55–65Gy) were most often performed. CFR was the most effective operation, with a 5 y DFS of 65%. A local recurrence rate of 29%, a regional recurrence rate of 16%, and a 17% rate of distant recurrence were reported. The OS and DFS at 5 years had mean values of 45% and 41%, and the OS at 10 years was 52%.

Additional data for OpS were extracted from publications in which results after TCR [18,20], CFR ± E-ass [7,8,9,11,12,17], or TFR ± E-ass [4,21] could be selectively analyzed. The mean follow-up periods ranged from 23 to 114 months, while follow-up periods ranged from 2 to 330 months. Advanced tumor stages were present in 49–100% of TCRs or CFRs, in contrast to 15.4–46.2% of TFRs. No relevant data were available regarding Hyams grading or Ki-67. If reported, NMs were achieved in 67.6–100% of cases, and postoperative complications were observed in 11.9–37.5%. RT was performed in 53.8–100% of all cases. Recurrences were observed in 15.4–61.5%. Crude OS, DFS, and DSS rates were 50–92.3%, 50–76.9%, and 75–92.3%, respectively. The 5 y OS rates were 61–77.7%, 5 y DFS 41.7–73.5%, 5 y DSS 77–82.6%, and 5 y RFS 64.2%. Where reported, the 10 y OS rates were 42–67.8%, 10 y DFS 57.1%, and 10 y DSS 53%.

Several studies compared ES and OpS [15,25,26,27,28,29]. The present study includes results after ES and OpS for ENB over a follow-up period of 40 years. Tumor stage was the only parameter that showed significant differences between the ES and OpS groups. Significantly more tumors with lower stages relative to all relevant tumor classifications were treated in the ES group in comparison with the OpS group (Table 1 and Table 2a). This is in line with experience reported in the literature. It has been reported that significantly more Kadish C/D tumors were operated on with OpS, but significantly more Kadish A/B tumors with ES [25,26,27]. The review by Devaiah et al. included 23 studies, comprising 361 patients, published from 1992 to 2008. The study states that survival after ES was significantly better in comparison with OpS (100% vs. ≈45%) or E-ass OpS (100% vs. ≈50%), even after the results were stratified according to the publication year. The median follow-up periods were similar in the ES and OpS groups (54.5 vs. 51.0 months) [25]. However, purely ES can also provide complete resection in more advanced tumors without compromising survival, provided that limits are recognized and respected [12,15,26,29,39,48,49]. De Bonnecaze et al. evaluated 24 publications including 283 patients, and 15 cases of their own, and investigated the long-term results after treatment for advanced-stage ENB. The highest survival rates after surgery for advanced tumors were obtained after ES, including over the long-term course. The 5 y OS was 95.8% after ES, 62.5% after E-ass OpS, and 60.9% after OpS [28]. Harvey et al. reported results from six tertiary centers including 109 patients—67 patients treated with ES and 42 with OpS (CFR ± E-ass). In comparison with ES, Kadish C-stage and high-grade tumors were more frequent in the OpS group (78.6% vs. 56.7% and 35.8% vs. 54.4%). ES was used to remove 53.1% of the Kadish C tumors [15]. A study by Barinsky et al. included 533 patients from the United States National Cancer Database; 51.8% underwent OpS and 48.2% ES. Among the tumors operated on with ES, 53.2% had Kadish C/D stages. In the ES group, the 5 y OS was higher in comparison with OpS (81.9% vs. 75.6%) [29].

Surgery with NMs was assigned greater importance than the surgical approach selected in some reports [12,15,27,29,49]. In the present study, NMs were obtained more often after ES in comparison with OpS (86.7% vs. 64.3%), probably due to the larger number of smaller tumors resected with ES. According to the review by Komotar et al. (including 47 studies and 453 patients), gross tumor resection was achieved in 98.1% in the ES group in comparison with 81.3% after CFR and 100% after TCR. NMs were obtained in 93.8% of cases after ES, 77.3% after CFR, and 95.8% after TCR [26]. Harvey et al. reported that NMs were obtained significantly more often after ES in comparison with OpS (88.1% vs. 51.2%), particularly also after surgery for Kadish B/C tumors (90% vs. 71.4% and 84.2 vs. 53.1%) [15].

More high-grade tumors and tumors with a higher Ki-67 index were observed after OpS in comparison with ES in the present study, but with no significant differences. Data for the Hyams grade, although it is recognized as an important prognostic factor [30,31,37], were not mentioned in the majority of the reports. In comparison with the available data, the number of high-grade tumors in the present study was in the upper range (Table 2). The Ki-67 index was not mentioned in any of the publications included in this study, but it seems to be emerging as a prognostic factor in recent publications [38].

The development of occurrence of postoperative complications was more frequent after OpS (39.3% vs. 13.3%), but with no significant differences. It has been reported that postoperative complication rates were lowest after ES in comparison with CFR or TCR [26], or lower after ES in comparison with OpS (28.1% vs. 52.9%) [27]. In one review, perioperative mortality was only observed after CFR (3.2%.) [26].

Not surprisingly, administration of (neo)adjuvant therapy RT/chemoradiotherapy was more often indicated after OpS (82.1%) than with ES (60%, Table 2), but no significant differences were observable in our study (Table 2b). Our data confirm the results reported by Harvey et al. (95.2% vs. 77.6%) [15] and Fu et al. (85 vs. 81.4%) [27]. According to Komotar et al., (neo)adjuvant RT was administered in 99.8% of cases after CFR, in 78.4% after TCR, and adjuvant RT in 77.3% after ES [26]. However, it must be noted that rates of (neo)adjuvant RT in the literature are up to 100% not only after OpS, but also after ES, reflecting the fact that advanced tumors are also resected with ES [15,23,24,26,27,40,41,42,43,44,45,46,47].

The development of or number of recurrences were lower and the mean time to first recurrence was higher after ES (26.7%; 86 months) in comparison with OpS (42.9%; 65 months) (Table 2b). The literature data show that local and regional recurrence rates are lowest after ES (8.0% and 6%) in comparison with CFR (22.1% and 17.3%) and TCR (16.7% and 8.3%), but also the mean time to local recurrence (33 vs. 42.2 vs. 59 months) [26]. In a review assessing 36 studies including 609 patients, Fu et al. reported that the frequencies of locoregional recurrence (17.4% vs. 45%) and distant metastases (1.1% vs. 7.5%) were significantly lower after ES in comparison with OpS [27].

The actuarial mean/median follow-up period was shorter after ES (139/109 months, range 9–494 months), but not significantly different from the OpS group (174/129 months, range 4–440 months) in the present study (Table 2b), and it was well above the periods published in the literature for both groups. In the review by Komotar et al., the mean follow-up periods after CFR, TCR, and ES were 71, 43.1, and 52 months, respectively [26]; according to Fu et al., the median follow-up after OpS was 43 months (range 1–312 months) and after ES 32.5 months (3–147 months) [27].

Survival data for a long-term follow-up period of more than 40 years were obtained in the present study. Crude survival and Kaplan–Meier survival rates for OS, DSS, and DFS for 10 years, 20 years, and 35 years were all better for patients after ES, but did not differ significantly between ES and OpS. A trend toward significantly better survival was observable for the 10 y, 20 y, and 35 y DSS, according to Kaplan–Meier, after ES (100% vs. 77.5%; *p* = 0.071), but not for DFS (55.9% vs. 35.3%, *p* = 0.523, Table 3, Figure 1 and Figure 2). This could be explained best by the significantly lower tumor stages, while no significant differences were notable regarding the presence of more aggressive tumors (high-grade, Ki-67 > 10%), although these were less often present in the ES group. In comparison, in the review by Komotar et al. at last follow-up (variable times), 63.4% of the patients were alive with no evidence of disease after ES, 61.6% after CFR, and 81.8% after TCR. The Kaplan–Meier 10 y OS was highest after CFR and the 10 y tumor progression-free survival was highest after ES [26]. For patients who had undergone ES, Fu et al. calculated 5 y OS, DSS, locoregional control (LRC), and MFS rates of 100%, 100%, 79.5%, and 89.8% and 10 y OS, DSS, LRC, MFS rates of 100%, 100%, 69.6%, and 89.8%. Kadish C/D tumors and high-grade tumors were associated with significantly better OS after ES. For OpS, the 5 y OS, DSS, LRC, and MFS rates were 71.2%, 77.5%, 78.8% and 87.3% and the 10 y OS, DSS, LRC, and MFS rates were 57.0%, 72.7%, 61.7% and 84% [27].

Only a few studies have specifically investigated long-term experience over decades (>10 or even >20 years) relative to surgical approaches for CFR/OpS [7,8,9,12,17,20,50], but actuarial survival data for a maximum of 10 years for ES have been published to date [12,15]. For OpS, 10 y OS of 42–93% [7,17], 10 y DSS of 53–63% [7,15], 10 y DFS of 57.1% [12], 15 y OS of 83% [20], 15 y DFS of 82.6% [8], 20 y DSS of 66.4–81.2% [9,50], and 20 y DFS of 29.8% [50] have been reported. For ES, 5 y OS of 87–100% [44,46], 5 y DSS of 85–100% [15,45], 5 y DFS of 71–100% [11,46], 10 y OS of 92–100% [12,20], 10 y DSS of 63% [15], 10 y DFS of 75.6% [12], and 15 y OS of 83% [20] have been reported.

The present study compares 35 y actuarial survival data after ES and OpS for OS (48.1%/40.5%), DSS (100%/77.5%), and DSF (55.9%/35.3%). The results show not only that ES is a successful and effective approach in ENB, including in the long-term over decades. In comparison with ES, the corresponding actuarial survival data after OpS were all lower for 10, 20, and 35 years, the only significant different prognostic factor being higher tumor stages in OpS.

The following limitations of this study should be mentioned: it is a retrospective study from a single center, suggesting an institutional bias, and includes a limited number of cases. It is virtually impossible to change the retrospective study design in research on esthesioneuroblastoma, as was stated in an earlier report: “Definitive prospective studies comparing open craniofacial resection with purely endoscopic techniques will probably never be available, owing to the relative rarity of esthesioneuroblastomas and their proclivity for late recurrence” [49].

## 5. Conclusions

ES may be considered as the surgical method of first choice for ENBs in Kadish stages A/B, and may also be a possible alternative in carefully selected cases of advanced ENB [12,15,26,29,39,48,49]. Hyams grade [30,31,37] and Ki-67 index [38] had no significant influence on outcomes after ES compared to OpS in the present study, but were not investigated in most of the publications cited here and should be examined more intensively in the future.

## Figures and Tables

**Table 1:** Tumor stages based on the classifications of Kadish, Morita, Biller, Dulguerov and Calcaterra, Koka, and Resto in 43 patients with esthesioneuroblastoma at first presentation.

Classification Type	Kadish 1975 [32] (A–C)	Morita 1993 [33] (A–D)	Biller 1990 [35] (T1–T4)	Dulguerov and Calcaterra 1992 [4] (T1–T4)	Koka 1998(T1–T4) [34]	Resto 2000 (T1–T3) [36]
Criteria on which findings are based	Clinical + radiologic	Clinical + radiologic	Clinical + surgical	Clinical + radiologic	Clinical + radiologic	Clinical + surgical
A or T1 (%)	1 (2.3)	1 (2.3)	19 (44.2)	6 (14.0)	16 (37.2)	19 (44.2)
B or T2 (%)	23 (53.5)	26 (53.5)	22 (51.2)	19 (44.2)	5 (11.6)	22 (51.2)
C or T3 (%)	19 (44.2)	19 (44.2)	2 (4.7)	16 (37.2)	12 (27.9)	2 (4.7)
D or T4 (%)	–	0 (0)	0 (0)	2 (4.7)	10 (23.3)	–

**Table 2:** Table 2a: Epidemiologic data and preoperative tumor data (stages), stratified relative to the different types of curative surgery in 43 patients with esthesioneuroblastoma. Table 2b: Intra- and postoperative data and data referring to the treatment relative to the different types of curative surgery in 43 patients with esthesioneuroblastoma.

**a**				
**Type of Curative Surgery** **Parameter**	**All Operations** **(n = 43)**	**Endonasal Surgery** **(n = 15)**	**Open Surgery:** **Bifrontal/Transcranial (n = 25),** **Transfacial (n = 3)**	**Comparison of Groups:** **Mann–Whitney U Test/** **Pearson Chi-Squared Test** **(*p* Values)**
Gender				
m f	48.84% (21/43) 51.16% (22/43)	60% (9/15) 40% (6/15)	42.9% (12/28) 57.1% (16/28)	n.s. (0.347) ^§^
Age (years)	52 ± 2.33 (M 52.0, R 15–84)	55.6 ± 3.49 (M 56; R 27–80)	50.25 ± 3.05 (M 51.5; R 15–84)	n.s. (0.256) ^+^
Age (years) < 50 (n, %) >50 (n, %)	32.56% (14/43) 67.4% (29/43)	20% (3/15) 80% (12/15)	39.3% (11/28) 60.7% (17/28)	n.s. (0.308) ^§^
Mod. Kadish/Morita A/B C/D	55.8% (24/43) 44.2% (19/43)	86.7% (13/15) 13.3% (2/15)	39.3% (11/28) 60.7% (17/28)	0.004 ^§^
Mod. TNM Dulguerov/Calcaterra				
T1/2 T3/4	58.1% (25/43) 41.9% (18/43)	86.7% (13/15) 13.3% (2/15)	42.9% (12/28) 57.1% (16/28)	0.009 ^§^
Mod. TNM Koka T1/2 T3/4	51.16% (22/43) 48.84% (21/43)	80% (12/15) 20% (3/15)	35.7% (10/28) 64.3% (18/28)	0.01 ^§^
Mod. Biller/Resto T1 T2 T3	44.2% (19/43) 55.16% (22/43) 4.65% (2/43)	86.7% (13/15) 13.3% (2/15) -----	21.4% (6/28) 71.4% (20/28) 7.1% (2/28)	0.0001 ^§^
Primary c/pN+	0% (0/43)	0% (0/15)	0% (0/28)	-----
**b**				
**Type of Curative Surgery** **Parameter**	**All Operations** **(n = 43)**	**Endonasal Surgery** **(n = 15)**	**Open Surgery:** **Bifrontal/Transcranial (n = 25),** **Transfacial (n = 3)**	**Comparison of Groups:** **Mann–Whitney U Test/** **Pearson Chi-Squared Test** **(*p* Values)**
Resection status				
Negative margins (n, %) Unknown/close/positive margins (n, %)	72.1% (31/43) 27.9% (12/43)	86.7% (13/15) 13.3% (2/15)	64.29% (18/28) 35.71% (10/28)	n.s. (0.164) ^§^
Complications after surgery	25.6% (11/43)	13.3% (2/15)	32.14% (9/28)	n.s. (0.308) ^§^
Hyams grading				n.s. (0.739) ^§^
		
I–II (n, %)	53.5% (23/43)	60% (9/15)	50% (14/28)	
	57.5% (23/40) #	64.3% (9/14) #	53.8% (14/26) #	
			
III-IV (n, %)	39.5% (17/43)	33.3% (5/15)	42.9% (12/28)	
	42.5% (17/40) #	35.7% (5/14) #	46.2% (12/26) #	
Ki-67 index (n, %)	13.24 ± 1.72 (M 10.15, R 0.26–40)	10.21 ± 2.31 (M 8; R 0.26–25)	14.97 ± 2.32 (M 15; R 0.57–40.2)	n.s. (0.214) ^+^
Ki-67-Index				n.s. (0.310) ^§^
		
<10% (n, %)	34.9% (15/43)	46.7% (7/15)	28.6% (8/28)	
	41.7% (21/36) *	53.8% (7/13) *	34.8% (8/23) *	
			
>10% (n, %)	48.8% (21/43)	40% (6/15)	53.6% (15/28)	
	58.3% (21/36) *	46.2% (6/13) *	65.2% (15/23) *	
Surgery only (n, %)	25.6% (11/43)	40% (6/15)	17.85% (5/28)	n.s. (0.15) ^§^
Pre-/postoperative RT (n, %)	74.4% (32/43)	60.0% (9/15)	82.14% (23/28)	n.s. (0.15) ^§^
Postoperative RT dose (Gy)	57.27 ± 1.77 (M 59.5; R 24–68)	58.48 ± 4.61 (M 63.35; R 24–66.6)	57.25 ± 1.60 (M 58; R 40–68.4)	n.s. (0.074) ^+^
Mono- vs. combined therapy				
Monotherapy Combined therapy	25.58% (11/43) 74.42% (32/43)	40% (6/15) 60% (9/15)	17.86% (5/28) 82.14% (23/28)	n.s. (0.15) ^§^
Multimodal therapy (S + RT ± ChT ± O ^¶^; n, %)	7.0% (3/43)	0% (0/15)	10.7% (3/28)	n.s. (0.098) ^§^
Recurrence/patient, any location (n, %)	37.2% (16/43)	26.7% (4/15)	42.9% (12/28)	n.s. (0.342) ^§^
Local recurrence/patient, (n, %)	13.95% (6/43)	6.66% (1/15)	17.85% (5/28)	n.s. (*p* = 0.403)
Time to first recurrence (months)	70.25 ± 18.77 (M 54.5, R 5–268)	86 ± 32.56 (M 92, R 7–153)	65 ± 23.12 (M 34.5, R 5–268)	n.s. (0.446) ^§^
Recurrences (number > 1)	11.63% (5/43)	6.7% (1/15)	14.3% (4/28)	n.s. (0.643) ^§^
Tumors with unfavorable factors	41.86% (18/43)	33.33% (5/15)	46.42% (13/28)	n.s. (0.523) ^§^
Follow-up time (months)	162 ± 21.54 (M 122; R 4–494)	139 ± 31.64 (M 109; R 9–494)	174.57 ± 28.54 (M 129; R 4–440)	n.s. (0.646) ^+^

^§^ Pearson chi-squared test with an exact 2-sided significance; ^+^ Mann–Whitney U-Test;. # Hyams grading was available in 93% of all cases (40/43); * Ki-67 index was available in 83.72% of all cases (36/43); ^¶^ O, others.

**Table 3:** Outcome and survival data: crude survival and survival estimates using the Kaplan–Meier method in 43 patients with esthesioneuroblastoma: comparison of endoscopic surgery and open surgery relative to the 10-year, 20-year, and 35-year overall survival (OS), disease-specific survival (DSS), and disease-free survival (DFS).

Type of Curative Surgery Survival	All Operations (n = 43)	Endoscopic Surgery (n = 15)	Open Surgery TCR (n = 25), TFR (n = 3)	Statistic Test (*p*-Values)
**Crude Survival**			**Pearson**	
**Chi-**				
**Squared Exact**				
**Test**				
Status alive dead	58.14% (25/43) 41.86% (18/43)	73.33% (11/15) 26.67% (4/15)	50.0% (14/28) 50.0% (14/28)	n.s. (0.199)
Survival status AND AWD DAD DOD	46.51% (20/43) 11.6% (5/43) 27.91% (12/43) 14.0% (6/43)	66.67% (10/15) 6.7% (1/15) 26.67% (4/15) 0% (0/15)	35.7% (10/28) 14.3% (4/28) 28.6% (8/28) 21.4% (6/28)	n.s. (0.133)
OS	58.1% (25/46)	73.33% (11/15)	50.0% (14/28)	n.s. (0.199)
DSS	86% (37/43)	100% (15/15)	78.6% (22/28)	n.s. (0.076)
DFS	58.14% (25/43)	66.7% (10/15)	53.57% (15/28)	n.s. (0.523)
**Kaplan–Meier Survival Estimation**			**Log-Rank Test**	
**(ES vs. OpS)**				
**OS** 10 y	66.9%	72.2%	63.2%	n.s. (0.402)
20 y	50.5%	48.1%	48.6%	
35 y	43.3%	48.1%	40.5%	
Mean ± SD	287.73 ± 35.02	311.10 ± 70.76	248.40 ± 36.62	
(95% CI, months)	(219.08–356.37)	(172.41–449.79)	(176.63–320.18)	
Median ± SD	367.0 ± 117.44	214.0	213.0 ± 111.41	
(95% CI, months)	(136.82–597.18)	-----	(0.0–431.36)	
**DSS** 10 y	84.9%	100%	77.5%	n.s. (0.071)
20 y	84.9%	100%	77.5%	
35 y	84.9%	100%	77.5%	
Mean ± SD	421.91 ± 27.18	-----	-----	
(95% CI, months)	(368.63–475.19)	-----	-----	
Median ± SD	-----	-----	-----	
(95% CI, months)	-----	-----	-----	
**DFS** 10 y	59.0%	55.9%	60.6%	n.s. (0.402)
20 y	50.6%	55.9%	42.4%	
35 y	44.3%	55.9%	35.3%	
Mean ± SD,	266.77 ± 38.25	311.25 ± 71.32	229.11 ± 38.53	
(95% CI, months)	(191.75–341.75)	171.47–451.03	(153.59–304.62)	
Median ± SD,	268.0 ± 97.64	-----	189 ± 57.40	
(95% CI, months)	(76.63–459.37)	-----	(76.49–301.51)	

TCR, transcranial resection; TFR, transfacial resection.

## Data Availability

The datasets used and/or analyzed during the current study are available from the corresponding author on reasonable request.
